# Beyond the Leash: Exploring the Public Health Dimension of Facility Dog Handler Experiences in Military Graduate Education

**DOI:** 10.3390/ijerph23081071

**Published:** 2026-08-18

**Authors:** Katherine Selby, Paula Bellini, Daryl Thorne

**Affiliations:** 1 Department of Family Medicine, Uniformed Services University of the Health Sciences, 4301 Jones Bridge Rd, Bethesda, MD 20814, USA; daryl.thorne@usuhs.edu; 2 Henry M. Jackson Foundation for the Advancement of Military Medicine, Inc., Bethesda, MD 20817, USA; paula.bellini.ctr@usuhs.edu; 3 Department of Medicine, Uniformed Services University of the Health Sciences, 4301 Jones Bridge Rd, Bethesda, MD 20814, USA; 4 Office for Student Affairs, Uniformed Services University of the Health Sciences, 4301 Jones Bridge Rd, Bethesda, MD 20814, USA

**Keywords:** facility dogs, graduate education, military education, students, wellbeing

## Abstract

**Highlights:**

**Public health relevance—How does this work relate to a public-health issue?**

- Addresses growing public-health concern of stress, anxiety, and burnout among graduate and military-affiliated students, all of which are contributors to long-term mental-health status and reduced academic performance.
- Examines the incorporation of facility dog programs as a non-pharmacological, community-based intervention to promote mental-health resilience and social connectedness, which are key determinants of public health.

**Public health significance—Why is this work of significance to public health?**

- Highlights the lived experiences of student facility dog handlers, a population responsible for mental-health interventions that remains understudied.
- Expands the understanding of how structured animal-assisted programs can serve as responsible public-health strategies with the goal of reducing stress-related illness and improving overall well-being in high-pressure educational environments.

**Public health implications—What are the key implications or messages for practitioners, policy makers and/or researchers in public health?**

- The long-term sustainability of facility dog programs requires ethical program development and institutional policies that explicitly address and mitigate the role conflict, caregiving demands, and emotional labor placed on student handlers, ensuring the program supports both the broader student population and the individuals who sustain it.
- Future research must prioritize systematic, context-sensitive qualitative and longitudinal inquiry into the lived experiences, well-being, and professional identity formation of graduate student facility dog handlers, particularly within high-pressure and culturally unique environments like military-affiliated graduate education.

**Abstract:**

Facility dogs are increasingly incorporated throughout higher-education institutions as integrative conduits for supporting students by reducing stress, anxiety, and emotional exhaustion. While these programs are designed to enhance student well-being, little is known about the experiences of the handlers who train, care for, and work with these dogs—specifically within military-affiliated programs. The handlers’ role necessitates a unique dynamic of caregiving, emotional labor, and professional responsibility and management that can create role conflict and additional stress among those graduate students who dedicate their time to the working dogs. Despite the growing presence of animal-assisted interventions in university settings, handler experiences in military graduate contexts are largely unexplored. In an effort to establish ethical and sustainable animal-assisted interventions and facility dog programs that contribute to population-level mental-health resilience and institutional public health, there must be greater understanding of the demands and implications of the handler’s workload to develop sustainability. Therefore, this paper highlights the need for qualitative inquiry to explore the intersection of handler experience and educational responsibilities, as integrated into the military environment.

## 1. Introduction

Animal-assisted interventions (AAIs) have gained attention as complementary approaches in supporting mental health and well-being across healthcare and educational settings [1,2]. AAI is an umbrella term that encompasses animal-assisted therapies (AATs), animal-assisted education (AAEs), and animal-assisted activities (AAAs), each differing in structure, goals, and professional oversight [3,4]. AATs are more structured and goal-oriented interventions requiring licensed professionals and documentation of client progress, whereas AAEs are used in educational settings and led by professionals to promote the development of essential skills. Facility dogs represent a broader framework of AAEs within the educational setting. In contrast, AAAs are often less structured, led by volunteers and do not require patient documentation [5]. The incorporation of facility dogs throughout higher education has been expanding as universities start to recognize the growing need for non-pharmacological interventions caused by the rising levels of stress, anxiety, and emotional exhaustion among graduate students [6,7].

The Uniformed Services University of the Health Sciences (USUHS) is a military-affiliated graduate institution that has incorporated facility dogs into their educational environment for over six years. Within military graduate education at USUHS, medical, graduate, and advanced practice nursing students not only navigate the pressures of graduate education, but also the cultural expectation, identity contingencies, and performance norms that are associated with military training. This presents a unique opportunity to examine the role of facility dogs and their student handlers within the complexities of military culture and graduate school education.

Despite the growing presence of facility dogs in university settings, there is limited research that focuses on the handlers’ lived experience. To the best of our knowledge, there is no research on facility dog handler experiences in military-affiliated graduate programs. By understanding how student handlers interpret and navigate their social interactions and daily care of the dogs, universities can develop ethical and sustainable facility dog programs that support both student well-being and canine welfare.

## 2. Literature Review

### 2.1. Facility Dogs in Clinical Settings

Facility dogs hold a unique and highly specialized position. They are specifically trained to perform skilled tasks tailored to the needs of multiple clients or persons within their specific environment—in this case, graduate students. The facility dog’s work requires advanced training to be able to respond appropriately in a variety of situations, thus ensuring they actively contribute to therapeutic, educational, or supportive services within the facility they serve [8]. The dogs are structurally integrated into the facility’s daily routines, which distinguishes them from visiting volunteer-led therapy dogs and positions them as part of the institution.

Research across healthcare and higher-education environments consistently demonstrates that brief, structured interactions with facility dogs can reduce stress and anxiety, improve mood, and enhance overall well-being among students [9,10,11,12]. Recent evidence suggests that these programs also act as a physiological “buffer” against future stressors. For instance, interacting with a therapy dog prior to a stressful task has been shown to attenuate physiological stress reactivity, specifically as measured by electrodermal activity [13]. Beyond immediate physiological relief, these interactions are linked to broader cognitive and academic benefits. Research indicates that the stress-reduction potential of human–canine interaction can lead to downstream improvements in attention, such as a reduction in mind-wandering and an increase in cognitive task performance—benefits that are critical for managing the heavy cognitive load of graduate studies [14].

Neurophysiological research provides further depth to these outcomes. Findings from EEG studies indicate that human–canine interactions can induce an increase in the alpha power spectrum in the prefrontal and frontal lobes—brain activity patterns associated with heightened relaxation, emotional stability, and improved concentration [15,16]. These effects are supported by physiological evidence showing decreased sympathetic activation and increased oxytocin release—mechanisms associated with emotional regulation and social bonding [17,18,19,20]. In clinical and pediatric contexts, facility dog programs have also been linked to improved morale and reduced burnout among professionals and patients [21,22,23], suggesting that their benefits extend beyond individual recipients to influence broader organizational well-being. Such improvements in social connectedness and belonging align with public-health determinants of mental-health resilience and community well-being.

This process may be further developed in a military-affiliated graduate program due to the pressures of cultural norms. In this case, the dog may serve as a personal support system as well as a relational bridge to how student handlers engage with their position within an academic and military environment. Research examining the experiences of dog handlers in pediatric settings indicates that these handlers’ express feelings of purpose, connection, and grounding, as well as time management, role conflict, and dog welfare concerns [21,24,25]. Few studies have sought to explore trained medical and graduate student dog handlers in military education. Building on this evidence, the following section describes the structure and implementation of the Uniformed Services University of the Health Sciences Facility Dog Program, which provides a practical context for examining how these benefits translate into daily academic life and handler experience within a military-affiliated educational environment.

### 2.2. The USU Facility Dog Program

The aim of developing a facility dog program at USUHS was to broaden access to animal-assisted activities (AAAs) and animal-assisted education (AAEs) across the university and to increase overall positive impact on students, faculty, and staff. The scope of existing resources and their effectiveness were enhanced through this program, which is housed within the F. Edward Hébert School of Medicine’s Office for Student Affairs Well-Being Program. Establishing a dedicated facility dog presence created consistent opportunities for support, comfort, and engagement within the campus community. To implement the program, an Administrative Instruction (AI) was modeled after and developed in consultation with the Walter Reed National Military Medical Center (WR AI 3000.07) and the USUHS School of Medicine’s Office for Student Affairs Well-Being Program.

The structure of the program was designed to designate one individual as the dog’s owner, responsible for housing, feeding, and medical care, while both students and staff volunteered to be trained. As the program matured, it grew to include additional dogs and owners, further extending its reach and impact across USUHS. It was anticipated, at the time of implementation, that their presence would provide students with healthy opportunities to take study breaks, decompress, and manage stress. Therefore, the overarching goals of the USUHS Facility Dog Program became twofold: (1) experiential—community building, emotional support, and stress reduction—and (2) educational—animal-assisted education as part of medical and graduate school orientation and the dogs’ roles within each.

The implementation and growth of the USUHS Facility Dog Program illustrate how structured animal-assisted initiatives can be embedded within an academic institution to promote well-being and community engagement. Beyond their immediate educational and emotional benefits, these programs also intersect with broader public-health concerns related to stress, mental-health resilience, and equitable access to support. The following section situates these institutional outcomes within a public-health framework, emphasizing how ethical program design and handler support contribute to sustainable mental-health promotion across university populations.

### 2.3. Public-Health Relevance

This work provides insights on the prevalent public-health issue of chronic stress, anxiety, and frequent burnout in university students, which has negative effects on learning capacity, sleep quality, and emotional well-being [7,26,27]. For university students, although mental health is a risk factor for poor academic performance, just 22% of university students actually seek help from formal university resources [28]. This can stem from students’ reluctance to use services where they are required to disclose their problems in the setting of the university they attend due to perceived implications for their educational future. Ethical program design supports both human and animal welfare, aligning with public-health principles of equity, prevention, and community resilience. This emphasizes the need for preventative and non-stigmatizing interventions with a focus on fostering a sense of belonging in the university community [29].

Future research examining student handlers would fill a knowledge gap that exists in program sustainability and long-term effectiveness of facility dog programs. Both of these factors depend on ethical development of the programs and explicit institutional policies. It is also important to consider caregiving demands, role conflict, and emotional labor of student handlers [21,22,24,25]. Knowing the lived experiences of student handlers is critical to designing programs that aid the general student population and those supporting them. Such programmatic use ensures that facility dog initiatives are able to remain a viable, ethically based, and sustainable form of mental-health support during high pressure and culturally unique environments like military-affiliated graduate education programs. Understanding these institutional and public-health dimensions provides essential context for examining the unique stressors faced by graduate students—particularly those enrolled in military-affiliated programs—where academic rigor and cultural expectations converge to shape mental-health outcomes and coping demands.

## 3. Graduate Student Stress

Graduate students frequently report high levels of anxiety and emotional exhaustion while balancing rigorous coursework, research demands, and performance expectations [9]. Among military-affiliated graduate students, these challenges are intensified by the dual expectations of academic excellence and adherence to military cultural norms. The need to maintain composure and competence across both domains can heighten stress and risk of burnout, underscoring the importance of early, preventative interventions within graduate education.

Evidence from higher-education settings demonstrates that structured facility dog programs can reduce self-reported anxiety during high-stress periods [11,30,31]. These effects are linked to physiological mechanisms—decreased sympathetic activation, increased oxytocin release—and to the availability of non-judgmental companionship that supports emotional regulation [4,20]. Beyond individual stress reduction, facility dogs can foster social connection and belonging [12], which can further enhance classroom engagement and peer relationships. Such outcomes align with public-health priorities of resilience and community well-being.

However, graduate and medical students who serve as handlers assume additional responsibilities that may amplify existing stressors. Managing the dog’s welfare, training, and public visibility introduces new layers of emotional labor and time commitment. Within military-affiliated programs, these demands intersect with institutional expectations of discipline and service, creating a distinct form of role strain. Recognizing and addressing these pressures is essential, as chronic stress and burnout among graduate students contribute not only to academic decline but also to long-term mental-health morbidity and reduced workforce readiness.

## 4. Conceptual Framework

A phenomenological framework structures the focus on the lived experience of facility dog student handlers within an educational setting. The emphasis on first-person accounts captures the emotions, relationships, and identity shifts that shape how handlers interpret their roles. This approach is well-suited to exploring complex layers of responsibility, relational connection with the dog, and navigation of academic and military cultural expectations.

Within a public-health context, this framework recognizes that individual experiences of stress, caregiving, and emotional labor are not isolated. They reflect broader patterns of mental-health risk and resilience within high-pressure educational systems. Understanding how handlers manage these demands offers insight into institutional conditions that influence well-being, belonging, and workforce readiness. By connecting personal narratives to systemic factors such as institutional support and community connectedness, the framework positions the handlers’ well-being as a meaningful indicator of collective health.

## 5. Research Gap

Despite a growing focus on the use of animal-assisted interventions, little research has been conducted addressing the benefits of employing facility dogs in graduate schools. Available studies have most commonly examined facility dog encounters around students, patients, or community members and have little focus on those involved in training, management, and integration of dogs in daily academic or clinical routines. As a result, the professional and personal consequences of AAI on a facility dog handler remain opaque. This knowledge gap is particularly acute in military-affiliated graduate programs, where students must also balance the expectations of military culture with academics. Universities cannot begin creating ethical, sustainable, and supportive facility dog programs without developing an in-depth understanding of how student handlers conceive, interpret, and make meaning of their roles. Yet to the best of our knowledge, no research exists regarding facility dog programs in the context of military graduate education, and there has been no research considering how the handler’s role contributes to student well-being, sense of belonging, or professional identity.

## 6. Future Research

Additional studies should center on the lived experiences of facility dog handlers and the burden of emotional labor involved in caretaking responsibilities. It is suggested that the focus of these could be the exploration of conflicting roles and responsibilities. Qualitative research methodologies through phenomenology or interpretative phenomenology would be appropriate to uncover the subjective and nuanced aspects of the handlers’ experiences which cannot be captured through quantitative measures alone. Longitudinal studies would also be useful in studying the lived experiences of graduate student handlers over time and how this impacts academic performance, help-seeking behavior, and influence on professional identity formation.

Beyond research, practical applications are essential for translating these insights into institutional policy. Universities should develop standardized protocols for handler selection, training, and workload management, supported by clear ethical guidelines and welfare monitoring for both humans and animals. Integrating facility dog programs into broader campus-wellness frameworks can enhance mental-health resilience and community connectedness. Collaboration among student affairs offices, veterinary services, and mental-health professionals will ensure comprehensive oversight and equitable access. At the policy level, funding mechanisms and interdepartmental partnerships can sustain these programs as evidence-based public-health interventions that strengthen institutional well-being and workforce readiness. Together, these research directions and applied strategies establish a foundation for evaluating facility dog programs not only as therapeutic interventions but as institutional systems requiring ethical oversight, sustainability planning, and continuous assessment. These issues are explored further in the following discussion.

## 7. Discussion

Building on these proposed research directions and institutional applications, the discussion turns to the broader implications of facility dog programs within graduate and military-affiliated education. Facility dog programs show promise in lowering levels of stress, strengthening community ties, and enriching the graduate student experience. The responsibilities that graduate student handlers hold have unique emotional and logistical demands. However, these demands are not always well-defined—particularly among student groups within military-affiliated graduate programs. Although documented benefits for animal-assisted interventions have been positively demonstrated in the literature, little is known about the lived experiences of the handlers who train, care for, and work with these dogs. Without greater knowledge of how handlers perceive and perform their positions, universities might potentially introduce programs that help educate the general student population at the risk of inadvertently placing a strain on the people who maintain them.

While facility dog programs can reduce stress and foster social connectedness, they also present challenges and unintended consequences that institutions must acknowledge and manage. Student handlers may experience increased caregiving demands, scheduling conflicts, and emotional labor, which can contribute to fatigue and impaired academic performance. Concurrently, animal welfare requires systematic safeguards to prevent canine stress in busy campus environments. These safeguards include regular rest periods, veterinary oversight, and behavioral monitoring. Institutional responsibility, therefore, includes clear role definitions, equitable distribution of duties, and transparent processes for selection, compensation, and conflict resolution. To mitigate these risks, programs should embed protections into their design, such as defined and limited handler hours, mandatory training on boundary setting and emotional labor, accessible mental-health, and peer-support resources. Formal animal-welfare protocols should include contingency plans and routine evaluation metrics that monitor handler well-being, academic outcomes, dog welfare indicators, and participation equity. Framing these measures as integral to program design helps ensure facility dog initiatives are ethically grounded, sustainable, and protective of both human and animal participants.

## 8. Conclusions

This paper highlighted the need for systematic and contextually appropriate studies on handler welfare, role conflict, and emotional labor within facility dog programs. Framing these initiatives through a public-health lens underscores their potential as sustainable, non-pharmacological strategies for promoting mental health, resilience, and community well-being in military-affiliated education. To translate this potential into practice, universities should adopt structured policies that define handler roles, limit caregiving hours, and provide access to mental-health and peer-support resources. Regular evaluation of handler well-being, academic performance, and animal welfare should be embedded into program oversight to ensure ethical and sustainable implementation. It is hoped that as students transition into their professional careers, their exposure to AAI through facility dog programs will positively impact their clinical practice and public-health implications.

For policymakers and institutional leaders, facility dog programs should be integrated into broader campus-wellness frameworks with clear standards for training, supervision, and animal-care accountability. Establishing interdepartmental collaboration—linking student affairs, veterinary services, and mental-health programs—can strengthen program sustainability and equity. Funding mechanisms that support handler training and veterinary care will further safeguard both human and animal participants. By integrating these measures into institutional policy, facility dog programs can evolve from ad hoc wellness initiatives into evidence-based public-health interventions that enhance resilience and belonging across university communities.

Taken together, these recommendations move facility dog programs beyond the leash—from individual acts of care to structured, ethically grounded public-health strategies that strengthen resilience, belonging, and well-being across university communities.

## Data Availability

No new data were created or analyzed in this study. Data sharing is not applicable to this article.
